# Prognostic gene expression analysis in a retrospective, multinational cohort of 155 multiple myeloma patients treated outside clinical trials

**DOI:** 10.1111/ijlh.13691

**Published:** 2021-08-26

**Authors:** Yan‐Ting Chen, Erik T. Valent, Erik H. van Beers, Rowan Kuiper, Stefania Oliva, Torsten Haferlach, Wee‐Joo Chng, Martin H. van Vliet, Pieter Sonneveld, Alessandra Larocca

**Affiliations:** ^1^ SkylineDx Rotterdam The Netherlands; ^2^ Myeloma Unit Division of Hematology University of Turin Turin Italy; ^3^ MLL Munich Leukemia Laboratory Munich Germany; ^4^ National University Cancer Institute National University Health System Singapore City Singapore; ^5^ Department of Medicine Yong Loo Lin School of Medicine National University of Singapore Singapore City Singapore; ^6^ Cancer Science Institute of Singapore National University of Singapore Singapore City Singapore; ^7^ Department of Hematology Erasmus MC Cancer Institute Rotterdam The Netherlands

**Keywords:** gene expression profiling, multiple myeloma, prognostication

## Abstract

**Objectives:**

Typically, prognostic capability of gene expression profiling (GEP) is studied in the context of clinical trials, for which 50%‐80% of patients are not eligible, possibly limiting the generalizability of findings to routine practice. Here, we evaluate GEP analysis outside clinical trials, aiming to improve clinical risk assessment of multiple myeloma (MM) patients.

**Methods:**

A total of 155 bone marrow samples from MM patients were collected from which RNA was analyzed by microarray. Sixteen previously developed GEP‐based markers were evaluated, combined with survival data, and studied using Cox proportional hazard regression.

**Results:**

Gene expression profiling‐based markers SKY92 and the PR‐cluster were shown to be independent prognostic factors for survival, with hazard ratios and 95% confidence interval of 3.6 [2.0‐6.8] (*P* < .001) and 5.8 [2.7‐12.7] (*P* < .01) for overall survival (OS). A multivariate model proved only SKY92 and the PR‐cluster to be independent prognostic factors compared to cytogenetic high‐risk patients, the International Staging System (ISS), and revised ISS. A substantial number of high‐risk individuals could be further identified when SKY92 was added to the cytogenetic, ISS, or R‐ISS. In the cytogenetic standard‐risk group, ISS I/II, and R‐ISS I/II, 13%, 23%, and 23% of patients with adverse survivals were identified.

**Conclusions:**

For the first time, this study confirmed the prognostic value of GEP markers outside clinical trials. Conventional prognostic models to define high‐risk MM are improved by the incorporation of GEP markers.

## 1 INTRODUCTION

Multiple myeloma (MM) is a heterogeneous hematologic malignancy which is considered incurable.^1^, ^2^, ^3^ Despite the significantly improved survival over the last decades, treatment responses remain diverse and not all patients benefit equally. This is especially the case for the 15%‐25% of high‐risk patients that suffer from rapid progression and poor survival. Various definitions of high‐risk disease are described in literature using different molecular and/or clinical variables, thereby typically identifying different patient subsets. Although most high‐risk definitions have been confirmed to correlate with poor outcome, their generalizability toward a "real‐world" scenario is often ambiguous, since 50%‐80% of the MM patients do not meet the strict trial eligibility criteria.^4^, ^5^, ^6^ For this reason, the question arises if the risk stratification markers developed in clinical trials settings can also be validated in MM patients treated outside trials.

Prognostication in MM is subject to change. In current practice, cytogenetic aberrations measured by fluorescence in situ hybridization (FISH) and the international staging system (ISS) are most common for MM prognostication.^7^, ^8^ Patients with at least one of the aberrations del(17p), t(4;14), or t(14;16) are commonly classified as cytogenetic (CA) high‐risk.^8^, ^9^ However, other descriptions are used as well.^10^ More recently, the revised ISS (R‐ISS) has been introduced to estimate risk of MM patients by extending the ISS with CA high‐risk and serum lactate dehydrogenase (LDH).^11^ There are, however, still a fair number of poor‐performing patients that are classified into low‐ or standard‐risk groups and vice versa.

Markers based on gene expression profiling (GEP) have shown promising results to predict clinical outcome. For example, SKY92—a biomarker that classifies MM patients into high‐ or standard‐risk based on the expression of 92 genes—has shown to correlate significantly with survival in multiple trial data sets.^12^, ^13^, ^14^, ^15^, ^16^, ^17^ This association turns out to be additive to conventional markers, because in combination with ISS or R‐ISS, SKY92 risk classification is further improved.^13^, ^18^, ^19^, ^20^ However, it has remained an open question whether these associations generalize to patients treated outside clinical trials.

In this retrospective, multinational study outside of clinical MM trials, we show that GEP‐based markers provide an accurate prognostic distinction between risk groups that better reflects survival, as compared to ISS, R‐ISS, and cytogenetics. Moreover, by combining the SKY92 classifier with conventional markers, clinical risk assessment of the MM patient can be improved.

## 2 MATERIALS AND METHODS

### 2.1 Cohort composition

Patients in this study were part of the Horizon 2020 funded MMpredict project, of which this current analysis included the subset of 155 patients treated outside clinical trials: 73 patients from the National University Health System, Singapore (NUHS), 37 from the Munich Leukemia Laboratory, Germany (MLL), and 45 from the University of Turin, Italy (UNITO). Patients aged 18 years or older and diagnosed with MM were included. Informed consent and ethical approval by institutional review boards were obtained, in accordance with the Declaration of Helsinki. Clinical information was obtained from patients' health records, including chromosomal abnormalities detected by interphase FISH for t(4;14), t(11;14), t(14;16)/t(14;20), del(17p), del(13q), and gain(1q) as reported by the local laboratories, and generally reflecting the FISH guidelines.^21^ Overall survival (OS; death of any cause) was collected for all patients. Progression free survival (PFS; disease progression or death from any cause) was available for samples from NUHS and UNITO. Both OS and PFS were truncated at 5 years.

### 2.2 Plasma cell purification and RNA isolation

Plasma cells were purified from bone marrow aspirates using CD138+‐based immunomagnetic bead selection (EasySep™; Stem Cell Technologies), stored in RLT buffer, and are only used if ≥80% CD38+ cells. RNA was isolated using a DNA/RNA AllPrep kit (QIAGEN; Hilden) according to the manufacturer's instructions. RNA concentration was measured using the NanoDrop spectrophotometer (Thermo Fisher Scientific), and quality and purity were assessed by the RNA 6000 assay (Agilent 2100 Bioanalyzer; Agilent Technologies). A minimum of 100 ng total RNA was used as test input.

### 2.3 Gene expression profiling

Gene expression profiling data were generated at a central laboratory (SkylineDx). RNA processing, target labeling, and hybridization to gene expression arrays were performed on the Human Genome U133 Plus 2.0 platform (Thermo Fisher Scientific) following the MMprofiler™ protocol (SkylineDx).^12^, ^22^, ^23^ The MMprofiler™ assay reports the SKY92 risk classification, the MM clusters (CD1, CD2, CTA, HY, LB, MF, MS, Myeloid, NFKB, NP, PRL3, and PR),^24^, ^25^ and GEP‐based models to predict chromosomal aberrations commonly reported by interphase FISH (t(4; 14), t(11; 14), and t(14; 16)/t(14; 20)).^26^

### 2.4 Statistical analysis

Univariate and multivariate survival analyses were performed by the Cox proportional hazard model provided that the proportional hazard assumption was met based on weighted residuals (“survival” package v3.1‐8 in R‐3.6.0). Hazard ratio estimates were expressed relative to the lowest‐risk group and assessed by a two‐sided Wald test. Analysis of deviance was performed with the “stats” package (v3.6.3). *P*‐values below .05 were considered to be significant through this study.

## 3 RESULTS

### 3.1 GEP‐based biomarkers are prognostic for MM patients treated outside of clinical trials

The 155 MM patients, treated outside of clinical trial setting, had a median age of 66 years (Table 1). Most of them received an immunomodulatory drugs (IMiD; 25%), a proteasome inhibitor (PI; 48%), or a combination of both (15%). No correlation was found between treatment and survival (Table S3, overview of treatment analysis is summarized in Supporting Information).

**TABLE 1:** Patient demographic and disease characteristics

Median age, years (range)	66 (32‐90)
≤65, n (%)	77 (50%)
>65, n (%)	78 (50%)
Sex, n (%)	
Female	68 (44%)
Male	87 (56%)
Ethnicity, n (%)	
Caucasian	46 (30%)
Asian	57 (37%)
Other	15 (10%)
Not specified	37 (24%)
Disease stage n (%)	
Newly diagnosed MM	138 (89%)
Relapsed and refractory MM	17 (11%)
LDH, n (%)	
≤ ULN (upper limit of normal)	92 (59%)
> ULN	14 (9%)
Missing	49 (32%)
International Staging System stage, n (%)	
ISS I	20 (13%)
ISS II	32 (21%)
ISS III	49 (31%)
Missing	54 (35%)
Revised ISS, n (%)	
R‐ISS I	11 (7%)
R‐ISS II	60 (39%)
R‐ISS III	21 (13%)
Missing	63 (41%)
Cytogenetics, n/N (%)	
t(4;14)^a^	20/155 (13%)
t(11;14)^a^	29/155 (19%)
t(14;16)/t(14;20)^a^	10/155 (6%)
del(17p)	12/128 (9%)
del(13q)	49/136 (36%)
gain(1q)	16/56 (29%)
CA high‐risk	39/134 (29%)
Gene expression profiling, n (%)	
SKY92	
High‐risk	35 (23%)
Standard‐risk	120 (77%)
MM clusters	
CD1‐cluster	10 (6%)
CD2‐cluster	17 (11%)
CTA‐cluster	1 (1%)
HY‐cluster	37 (24%)
LB‐cluster	12 (8%)
MF‐cluster	11 (7%)
MS‐cluster	18 (12%)
Myeloid‐cluster	26 (17%)
NFKB‐cluster	7 (4%)
NP‐cluster	1 (1%)
PRL3‐cluster	6 (4%)
PR‐cluster	9 (6%)

^a^ GEP‐based results were used for patients whose chromosomal translocations were not performed or not available, (n = 38 t[4;14], n = 37 t[11;14], and n = 57 t[14;16]/t[14;20]).

Survival outcomes (median follow up of 31 months, using censored data only) did not correlate with either one of the three sites of inclusion (Figure S1). Compared to FISH, GEP‐based t(4;14), t(11;14), and t(14;16)/t(14;20) had both a positive and negative percent agreement (PPA, NPA) above 80% (Table S1) corroborating a previous study^27^ and therefore were considered equivalent in cases for which FISH was not performed or not available (Table 1).

**FIGURE 1:**
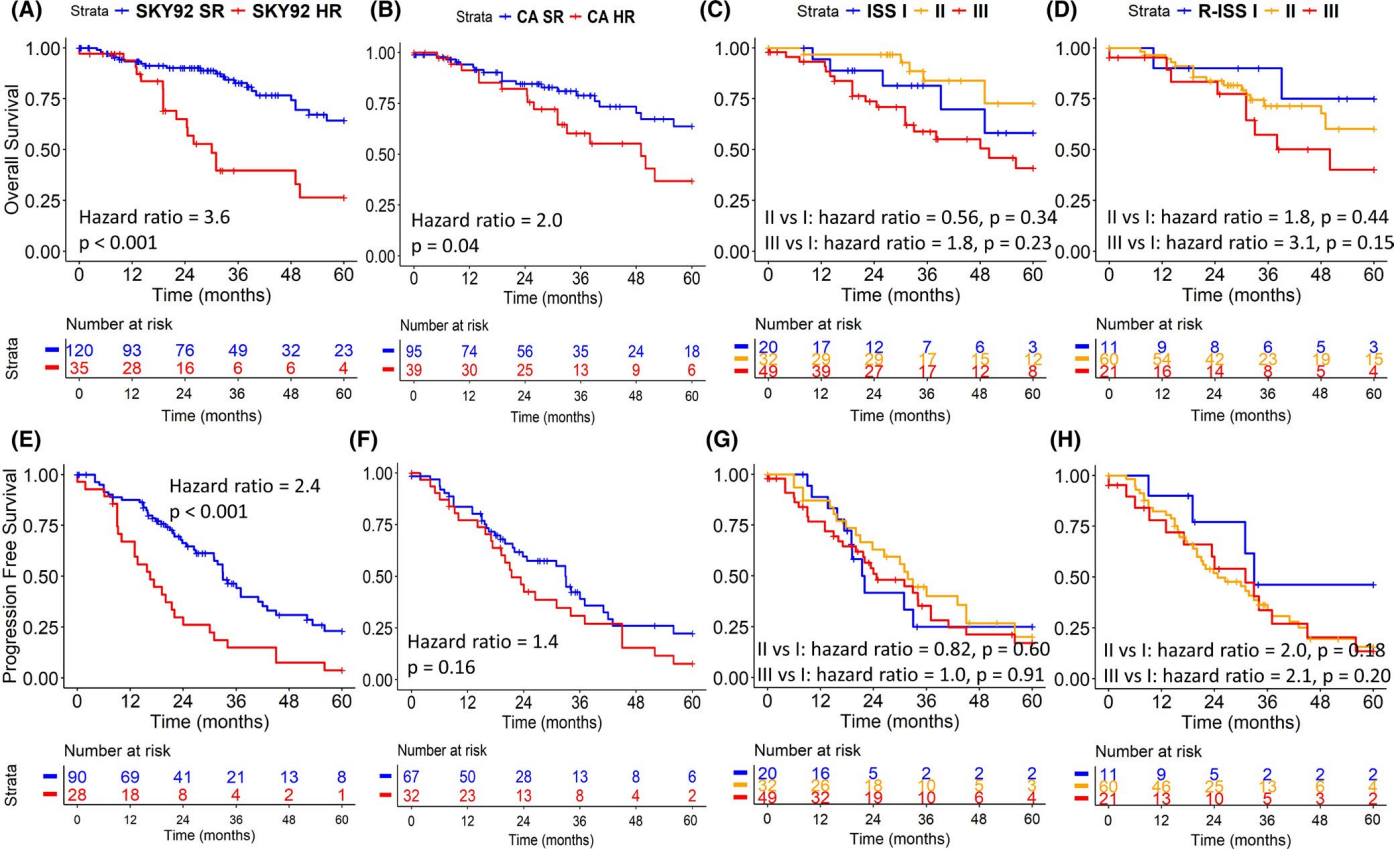
SKY92 (A, E) had larger hazard ratios with smaller *P*‐values for both overall survival (OS) (A‐D) and progression free survival (PFS) (E‐H) compared to frequently used stratification markers high‐risk cytogenetic aberrations (CA) (del(17p) + t(4;14) + t(14;16))(B, F), ISS (C, G), and R‐ISS (D, H)

SKY92 identified 23% of patients as high‐risk with a hazard ratio for OS (HR_os_) and PFS (HR_pfs_), and corresponding 95% confidence interval (CI) of HR_os_: 3.6 [2.0‐6.8] (*P* < .001) and HR_pfs_: 2.4 [1.5‐4.0] (*P* < .001; Figure 1A,E) in a univariate analysis. Out of all 12 gene expression clusters, the proliferation (PR) cluster had the strongest association with survival showing a HR_os_: 5.8 [2.7‐12.7] (*P* < .01) and HR_pfs_: 3.0 [1.4‐6.4] (*P* < .01), although its sample size of 9 patients was small (Table 2). The CA high‐risk definition of at least one of t(4;14), t(14;16), or del(17p)^8^—was positive for 39 out of 134 patients (29%)—and associated significantly for HR_os_: 2.0 [1.0‐3.9] (*P* = .04), but not for HR_pfs_: 1.4 [0.87‐2.4] (*P* = .16; Figure 1B,F). ISS staging was available for 101 of 155 patients (ISS I:II:III = 20:32:49). Strikingly, no significant differences were found between stage III versus I with HR_os_: 1.8 [0.7‐4.8] (*P* = .24) and HR_pfs_: 1.0 [0.5‐2.1] (*P* = .95; Figure 1C,G). Availability of LDH and CA restricted the data set further to 92 cases for the R‐ISS analysis (R‐ISS I:II:III = 11:60:21), resulting in a for stage III versus I, HR_os_: 3.1 [0.66‐14.4] (*P* = .15) and HR_pfs_: 2.0 [0.66‐6.1] (*P* = .22; Figure 1D,H).

**TABLE 2:** Survival analysis of OS and PFS analyzed by Cox proportional hazard model

	Risk factor	OS (n = 155)		PFS (n = 118)	
		HR (95% CI)	*P*	HR (95% CI)	*P*
GEP markers	SKY92 high‐risk	3.6 (2.0‐6.8)	<.01**	2.4 (1.5‐4.0)	<.01**
	CD1‐cluster	NA	NA^c^	0.91 (0.33‐2.5)	.85
	CD2‐cluster	NA	NA^b^	1.4 (0.60‐3.3)	.44
	CTA‐cluster	NA	NA^a^	NA	NA^a^
	HY‐cluster	0.79 (0.39‐1.6)	.53	0.99 (0.59‐1.7)	.97
	LB‐cluster	0.20 (0.03‐1.5)	.11	0.59 (0.25‐1.4)	.22
	MF‐cluster	1.6 (0.49‐5.2)	.44	1.7 (0.8‐4.0)	.20
	MS‐cluster	2.0 (0.97‐4.3)	.06	1.2 (0.65‐2.2)	.56
	Myeloid‐cluster	0.81 (0.32‐2.1)	.66	0.66 (0.30‐1.4)	.29
	NFKB‐cluster	0.58 (0.08‐4.2)	.59	0.71 (0.10‐5.2)	.74
	NP‐cluster	NA	NA^a^	NA	NA^a^
	PRL3‐cluster	0.51 (0.07‐3.7)	.51	NA	NA^b^
	PR‐cluster	5.8 (2.7‐12.7)	<.01**	3.0 (1.4‐6.4)	<.01**
Clinical markers/variables	t(4;14)	NA	NA^b^	1.06 (0.6‐1.9)	.84
	t(11;14)	0.4 (0.1‐1.1)	.08	1.2 (0.6‐2.2)	.58
	t(14;16)/t(14;20)	1.5 (0.47‐5.0)	.48	1.5 (0.6‐3.4)	.38
	del(17p)	1.6 (0.6‐4.0)	.37	1.8 (0.89‐3.7)	.11
	del(13q)	NA	NA^b^	NA	NA^b^
	gain(1q)	0.46 (0.06‐3.7)	.47	1.4 (0.50‐4.1)	.50
	CA high‐risk	2.0 (1.0‐3.9)	.04*	1.4 (0.87‐2.4)	.16
	ISS II vs I	0.55 (0.17‐1.8)	.32	0.82 (0.39‐1.7)	.59
	ISS III vs I	1.8 (0.68‐4.8)	.24	1.0 (0.51‐2.1)	.95
	R‐ISS II vs I	1.7 (0.40‐7.6)	.46	2.0 (0.72‐5.7)	.18
	R‐ISS III vs I	3.1 (0.66‐14.4)	.15	2.0 (0.66‐6.1)	.22
	LDH >ULN	1.7 (0.71‐3.8)	.24	0.97 (0.49‐1.9)	.94
	Age, in year	1.0 (0.97‐1.0)	.8	1.0 (0.98‐1.0)	.97

Note

Significant codes: **P < .01, *P < .05.

Abbreviation: NA, Not available; OS, Overall survival; PFS, Progression free survival.

^a^ CTA‐ and NP‐clusters both only consist of one sample (Table 1).

^b^ Violated the proportional hazard assumption.

^c^ Did not have any event in the positive group, for which survival analysis could not be performed.

In the subsequent multivariate analysis—using all significant univariate markers as input (SKY92, PR‐cluster, and CA high‐risk; Table S2), only SKY92 and the PR‐cluster remained independently significant with a HR_os_: 2.9 [1.2‐6.9] (*P* = .02), HR_pfs_: 2.3 [1.1‐4.6] for SKY92, and a HR_os_: 3.7 [1.5‐9.1] (*P* = .004), HR_pfs_: 2.5 [1.1‐5.8] (*P* = .04) for the PR‐cluster.

**FIGURE 2:**
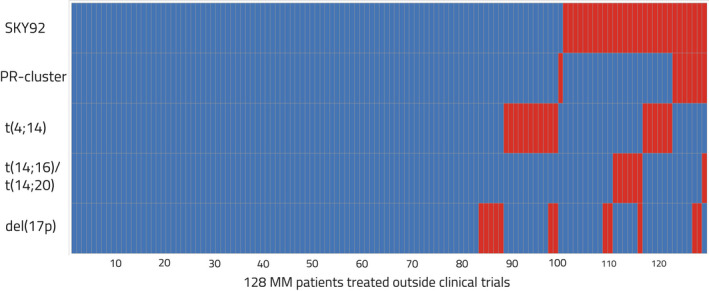
Classification and overlap between five prognostic high‐risk markers (SKY92, PR‐Cluster, t(4;14), t(14;16)/t(14;20), and del(17p)) in MM patients with data available for all five markers (n = 128). Together, a total of 46 individuals (36%) were identified as high‐risk and 82 patients (64%) as standard‐risk. The red color indicates the positive cases for each of the markers, and blue specifies the negative patients

In 128 patients with data available for both GEP‐based markers (SKY92 + PR) and the three high‐risk FISH markers, patients were identified as single (n = 23), double (n = 19), and triple (n = 4) hit disease (Figure 2). Among these 46 (36%) high‐risk patients, 13 (10%) were uniquely acknowledged by the GEP markers and were significantly correlated with adverse outcomes compared to those not identified by any of the five markers (HR_os_: 8.6 [3.5‐21.1], HR_pfs_: 6.3 [3.0‐13.3]), highlighting the added prognostic value of GEP markers. SKY92 overarched the classification of the PR‐cluster; 8 of 9 PR‐cluster patients were in the SKY92 high‐risk group, disabling the assessment of association with patients identified as PR‐positive and SKY92 standard‐risk (Figure S2). In the following analyses, we proceed with only SKY92 because it identified a larger proportion of individuals and it overlapped with the majority of PR‐cluster patients.

### 3.2 GEP‐based classifiers identify patients overlooked by other markers

In an analysis of variance, it was shown that adding SKY92 to the OS Cox models significantly improved all stratifications (*P* = .002, 0.0004, or 0.0003 for CA, ISS, or R‐ISS). On the other hand, the addition of CA, ISS, or R‐ISS to a model with SKY92 alone was only significant for ISS (*P* = .92, .04, and .92, respectively). For PFS, adding SKY92 to any of the existing markers always improved the model (*P* = .005, .002, and .006 for CA, ISS, and R‐ISS), while adding existing markers to SKY92 did not result in significant improvements (*P* = .54, .86, .46).

**FIGURE 3:**
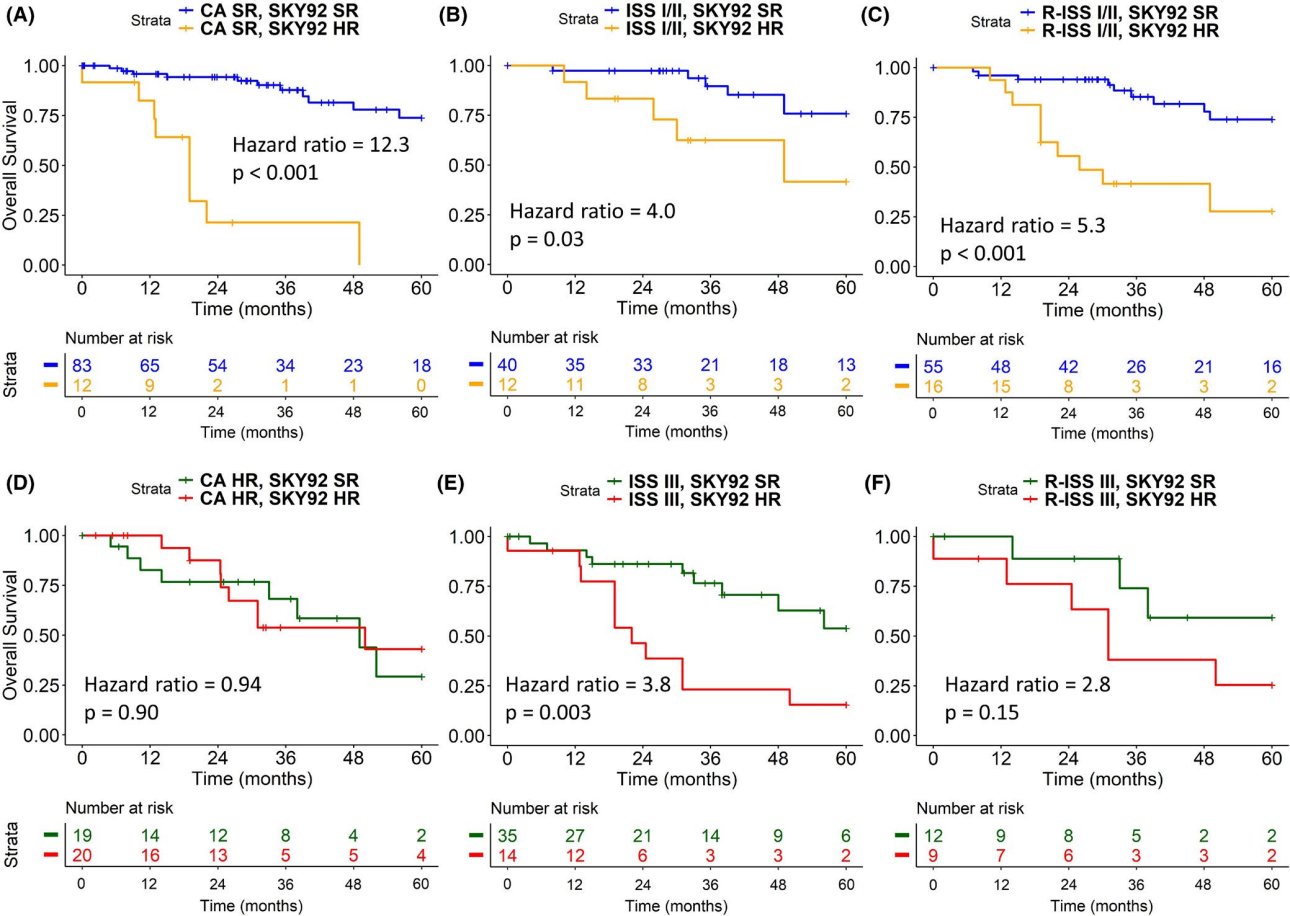
SKY92 identified patients with adverse overall survival (OS) in (A) cytogenetic aberrations (CA) standard‐risk, (B) ISS I/II, and (C) R‐ISS I/II. SKY92 also identified patients with favorable OS in (E) ISS III, but not in (D) CA high‐risk nor (F) R‐ISS III

SKY92 identified high‐risk patients classified as standard‐risk by CA (Figure 3A). Among these 95 standard‐risk patients, 12 (13%) were identified as SKY92 high‐risk with a HR_os_: 12.3 [4.8‐31.9] (*P* < .001) compared to the SKY92 standard‐risk group (n = 83, 87%). Within CA high‐risk patients, the 92‐gene classifier was not significant with a HR_os_: 0.94 [0.35‐2.5] (*P* = .90; Figure 3D). Similarly, SKY92 found 12 (23%) high‐risk patients out of 52 ISS I/II with a HR_os_: 4.0 [1.2‐13.2] (*P* = .03; Figure 3B) and 16 (23%) high‐risk patients out of 71 R‐ISS I/II with a HR_os_: 5.3 [2.1‐13.2] (*P* < .001; Figure 3C). Moreover, SKY92 was able to detect patients with adverse PFS in CA standard‐risk, ISS I/II and R‐ISS I/II (Figure S3A‐C). Furthermore, within the 49 patients with ISS stage III, only 14 (28%) were identified as SKY92 high‐risk with a HR_os_: 3.8 [1.5‐9.1] (*P* = .003; Figure 3E). Comparable observations were done for PFS (Figure S3).

Since it was shown that SKY92 is useful when added to other markers, we extended the CA high‐risk group by the addition of SKY92 and followed the previously proposed stratifications of combining SKY92 with ISS or R‐ISS (Figure S4).^13^, ^20^ By comparing the individual risk stratifications (Figure 1B‐D,F‐H) with those combined with SKY92, it is evident the combined stratifications all resulted in more extreme hazard ratios with decreased *P*‐values—confirming the added value of the GEP‐based classifier.

## 4 DISCUSSION

In the current study, we demonstrated that gene expression‐based analysis adds value to the prognostication of MM patients treated outside clinical trials. In the multivariate analysis, the SKY92 risk classifier and PR‐cluster remained independently associated with survival, in contrast to frequently used clinical risk indicators (ISS, R‐ISS, and CA). By incorporating GEP‐based classifiers, additional high‐risk patients were detected that were not identified by other markers. These high‐risk patients had associated adverse outcomes and could potentially be referred to clinical trials focusing on this subpopulation^28^ or could qualify for better monitoring and a more intense treatment.

Multiple studies have made it apparent that data from randomized controlled studies may not always reflect results for patients undergoing treatment in routine clinical practice.^5^, ^6^, ^29^ Due to a selection of younger and fit patients in clinical studies—reflecting the strict eligibility criteria—the MM population treated outside of clinical trials is enriched with comorbidities, inadequate organ function, and a lower performance status, which is frequently translated into poorer survival outcomes.^30^ In our cohort, the median PFS was 31 months for all combined treatments. This is longer compared to real‐world registries from Denmark and The Netherlands, who reported 18 months^31^, ^32^ and contrasted with recent phase 3 clinical data in which a median PFS over 40 months was described in the newly diagnosed setting.^33^, ^34^, ^35^

From the list of evaluated biomarkers, the association of SKY92 high‐risk and PR‐positive patients with poor OS and PFS had the strongest prognostic effect, demonstrating their accuracy and reliability in this cohort reflecting a "real‐world" scenario. Compared to frequently applied clinical risk indicators, both GEP‐based markers had more pronounced hazard ratios with smaller *P*‐values, potentially reflecting the new source of information (tumor gene expression) not captured by the other markers focusing on tumor genetics and more systemic host factors. These results support previous findings in patients in clinical trials.^18^

In contrast to conventional markers, gene classifiers better capture the global state of a tumor. For example, in a IGH translocation, a gene is juxtaposed the IGH‐locus and expected to become overexpressed. There might, however, be mechanisms counteracting the overexpression, such that the actual expression of the translocated (or co‐regulated) genes might be more informative. In addition, a single gene classifier is likely to target multiple prognostic mechanisms. It has been shown that the 92 genes in the SKY92 are enriched for the long arm of chromosome 1.^12^ A total of nine genes are located on 1q (Figure S6), of which S100A6 has been described in relation to 1q21 amplification in MM and other cancer types.^36^ Other known and possibly yet undiscovered cancer mechanisms are captured by the SKY92, including cell cycle pathways (BIRC5, TOP2A, and CENPE), cell growth and proliferation (FGFR3), DNA repair (FANCF, FANCI, POLQ), and transcription factor (STAT1). However, it is important to note that the genes within the signature reflect optimal performance of the signature rather than a biological definition of survival in MM.

In contrast to SKY92, which held up in this cohort as compared to its validation in trial patients published previously,^12^, ^13^, ^14^, ^15^, ^16^, ^17^ the prognostic value of ISS did not reach significance in our current cohort, as ISS I demonstrated intermediate risk relative to ISS II and III. This is unexpected, as several studies have shown ISS to be prognostic in other real‐world registries.^31^, ^32^ As we could not find a confounding factor (Table S4) nor registry errors, we assume this is a correct result, which might be biased due to the lack of information on a subset of subjects and/or the lower observed numbers in ISS I.

Because it is unavoidable that some patients with adverse survival will be classified into the SKY92 standard‐risk group and/or patients with favorable survival in the SKY92 high‐risk group, it proved useful to combine multiple prognostic markers to strengthen their prognostic power. Analysis of variance has shown that adding SKY92 to any of CA, ISS, or R‐ISS significantly improved the stratification for OS, while only addition of ISS to a model with SKY92 was significant, confirming one of our previous findings.^18^ By doing so, we have observed that cytogenetics with SKY92 could identify more high‐risk patients, and with a larger hazard ratio for OS compared to the single markers alone. Especially in situations where ISS was weakly associated with prognosis—like in this cohort for OS stratification—SKY92 helped to significantly distinct the stratification of MM patients in a three‐level classification. Moreover, the majority overlapping R‐ISS stages II and III were also discriminated when adding SKY92, resulting in more distinguished intermediate‐ and high‐risk groups.

To conclude, this multinational study in MM patients treated outside clinical trials recapitulates GEP‐based information as strongly prognostic for both OS and PFS. In addition, we found that the prognostic indications of CA, ISS, and R‐ISS are improved by the addition of SKY92, indicating the added clinical value of gene‐based technology to personalized management in MM patients.

## CONFLICT OF INTEREST

YTC, ETV, EHB, RK, and MHV are employees and option holders of SkylineDx BV. SO: Honoraria: Janssen, Takeda, Amgen. Advisory Board: Adaptive Biotechnologies, Amgen, Takeda, Janssen. TH is part owner of MLL Munich Leukemia Laboratory. WJC has no relevant conflict of interest to this work. PS: Takeda: Honoraria, Research Funding; Janssen: Honoraria, Research Funding; Karyopharm: Honoraria, Research Funding; Skyline: Research Funding; Celgene: Honoraria, Research Funding; Amgen: Honoraria, Research Funding. AL: Amgen: Honoraria, Bristol‐Myers Squibb: Honoraria, Membership on an entity's Board of Directors or advisory committees, Celgene: Honoraria, Membership on an entity's Board of Directors or advisory committees, Janssen: Membership on an entity's Board of Directors or advisory committees, Honoraria, Takeda: Membership on an entity's Board of Directors or advisory committees.

## AUTHOR CONTRIBUTIONS

The authors confirm contribution to the paper as follows: EHvB, MHvV, PS, and AL conceived the project and design. SO, TH, W‐JC, PS, AL, and the SkylineDx lab contributed to the collection of the data. Y‐TC, ETV, EHvB, RK, and MHvV performed the analysis and interpretation of initial results. Y‐TC and ETV drafted the manuscript. All authors reviewed and commented on the results and approved the final version of the manuscript.

## Supporting information

Supplementary Material

## Data Availability

The data that support the findings of this study are available from the corresponding author upon reasonable request.

## References

[1] Sonneveld P , Schmidt‐Wolf IGH , Van Der Holt B , et al. Bortezomib induction and maintenance treatment in patients with newly diagnosed multiple myeloma: Results of the randomized phase III HOVON‐65/ GMMG‐HD4 trial. J Clin Oncol. 2012;30(24):2946‐2955. 22802322 10.1200/JCO.2011.39.6820

[2] Kumar SK , Rajkumar SV , Dispenzieri A , et al. Improved survival in multiple myeloma and the impact of novel therapies. Blood. 2008;111(5):2516‐2520. 17975015 10.1182/blood-2007-10-116129 PMC2254544

[3] Sonneveld P , Goldschmidt H , Rosiñol L , et al. Bortezomib‐based versus nonbortezomib‐based induction treatment before autologous stem‐cell transplantation in patients with previously untreated multiple myeloma: a meta‐analysis of phase III randomized, controlled trials. J Clin Oncol. 2013;31(26):3279‐3287. 23897961 10.1200/JCO.2012.48.4626

[4] Rearden J , Hanlon AL , Ulrich C , Brooks‐Carthon M , Sommers M . Examining differences in opportunity and eligibility for cancer clinical trial participation based on sociodemographic and disease characteristics. Oncol Nurs Forum. 2016;43(1):57‐66. 26679445 10.1188/16.ONF.57-66 PMC5934287

[5] Klausen TW , Gregersen H , Abildgaard N , et al. The majority of newly diagnosed myeloma patients do not fulfil the inclusion criteria in clinical phase III trials. Leukemia. 2019;33(2):546‐549. EHA Library.215623; PS1323. 30267010 10.1038/s41375-018-0272-0

[6] Terpos E , Mikhael J , Hajek R , et al. Management of patients with multiple myeloma beyond the clinical‐trial setting: understanding the balance between efficacy, safety and tolerability, and quality of life. Blood Cancer J. 2021;11(2):40. 33602913 10.1038/s41408-021-00432-4 PMC7891472

[7] Greipp PR , Miguel JS , Dune BGM , et al. International staging system for multiple myeloma. J Clin Oncol. 2005;23(15):3412‐3420. 15809451 10.1200/JCO.2005.04.242

[8] Jacobus SJ , Kumar S , Uno H , et al. Impact of high‐risk classification by FISH: an Eastern Cooperative Oncology Group (ECOG) study E4A03. Br J Haematol. 2011;155(3):340‐348. 21902684 10.1111/j.1365-2141.2011.08849.x PMC3192237

[9] Moreau P , San Miguel J , Sonneveld P , et al. Multiple myeloma: ESMO clinical practice guidelines for diagnosis, treatment and follow‐up. Ann Oncol. 2017;28:iv52‐iv61. 28453614 10.1093/annonc/mdx096

[10] Sonneveld P , Avet‐Loiseau H , Lonial S , et al. Treatment of multiple myeloma with high‐risk cytogenetics: a consensus of the International Myeloma Working Group. Blood. 2016;127(24):2955‐2962. 27002115 10.1182/blood-2016-01-631200 PMC4920674

[11] Palumbo A , Avet‐Loiseau H , Oliva S , et al. Revised international staging system for multiple myeloma: a report from international myeloma working group. J Clin Oncol. 2015;33(26):2863‐2869. 26240224 10.1200/JCO.2015.61.2267 PMC4846284

[12] Kuiper R , Broyl A , de Knegt Y , et al. A gene expression signature for high‐risk multiple myeloma. Leukemia. 2012;26(11):2406‐2413. 22722715 10.1038/leu.2012.127

[13] van Beers EH , van Vliet MH , Kuiper R , et al. Prognostic validation of SKY92 and its combination with ISS in an independent cohort of patients with multiple myeloma. Clin Lymphoma Myeloma Leuk. 2017;17(9):555‐562. 28735890 10.1016/j.clml.2017.06.020

[14] Shah V , Sherborne AL , Johnson DC , et al. Predicting ultrahigh risk multiple myeloma by molecular pro fi ling : an analysis of newly diagnosed transplant eligible myeloma XI trial patients. Leukemia. 2020;34:3091‐3096. 32157174 10.1038/s41375-020-0750-z PMC7584474

[15] Wester R , van der Holt B , Asselbergs E , et al. Phase 2 study of carfilzomib, thalidomide, and low‐dose dexamethasone as induction/consolidation in newly diagnosed, transplant eligible patients with multiple myeloma, the Carthadex trial. Blood. 2016;128(22):1141. 10.3324/haematol.2018.205476 PMC6821616 30948492

[16] van Vliet M , Jasielec J , Dytfeld D , et al. Prognostic and Predictive Gene Expression Profiling (GEP) markers confirmed in carfilzomib, lenalidomide, and dexamethasone (KRd) treated newly diagnosed multiple myeloma (NDMM) patients (Pts). Blood. 2014;124(21):2141.

[17] Van BEH , Terragna C , Kuiper R , et al. MMprofiler with SKY92 combined with iss identifies high and low risk multiple myeloma in the VTD arm of gimema‐MMY‐3006. Blood. 2017;130:4358.

[18] Kuiper R , Van Duin M , Van Vliet MH , et al. Prediction of high‐ and low‐risk multiple myeloma based on gene expression and the International Staging System. Blood. 2015;126(17):1996‐2004. 26330243 10.1182/blood-2015-05-644039 PMC4616233

[19] van Vliet MH . The combination of SKY92 and ISS provides a powerful tool to identify both high risk and low risk multiple myeloma cases, validation in two independent cohorts. Blood. 2015;126:2970.

[20] Kuiper R , Zweegman S , van Duin M , et al. Prognostic and predictive performance of R‐ISS with SKY92 in older patients with multiple myeloma: The HOVON‐87/NMSG‐18 trial. Blood Adv. 2020;4(24):6298‐6309. 33351127 10.1182/bloodadvances.2020002838 PMC7756986

[21] Ross FM , Avet‐Loiseau H , Ameye G , et al. Report from the European myeloma network on interphase FISH in multiple myeloma and related disorders. Haematologica. 2012;97(8):1272‐1277. 22371180 10.3324/haematol.2011.056176 PMC3409827

[22] Gautier L , Cope L , Bolstad BM , Irizarry RA . Affy ‐ Analysis of Affymetrix GeneChip data at the probe level. Bioinformatics. 2004;20(3):307‐315. 14960456 10.1093/bioinformatics/btg405

[23] van Beers EH , Huigh D , Bosman L , et al. Analytical validation of SKY92 for the Identification of high‐risk multiple myeloma. J Mol Diagnostics. 2021;23(1):120‐129. 10.1016/j.jmoldx.2020.10.010 33152501

[24] Broyl A , Hose D , Lokhorst H , et al. Gene expression profiling for molecular classification of multiple myeloma in newly diagnosed patients. Blood. 2010;116(14):2543‐2553. 20574050 10.1182/blood-2009-12-261032

[25] Zhan F , Huang Y , Colla S , et al. The molecular classification of multiple myeloma. Blood. 2006;108(6):2020‐2028. 16728703 10.1182/blood-2005-11-013458 PMC1895543

[26] Van Vliet M , Kuiper R , Broijl A , et al. An assay for simultaneous diagnosis of t(4;14), t(11;14), t(14;16)/t(14;20), del1p, add1q, del13q, del17p, MS/MF expression clusters, and the SKY‐92 high‐risk signature in multiple myeloma patients. Haematologica. 2013;98 (s1)(101):abstract n. P234.

[27] Hose D , Beck S , Salwender H , et al. Prospective target assessment and multimodal prediction of survival for personalized and risk‐adapted treatment strategies in multiple myeloma in the GMMG‐MM5 multicenter trial. J Hematol Oncol. 2019;12(1):1‐12. 31242924 10.1186/s13045-019-0750-5 PMC6595705

[28] Brown S , Sherratt D , Hinsley S , et al. MUK nine OPTIMUM protocol: a screening study to identify high‐risk patients with multiple myeloma suitable for novel treatment approaches combined with a phase II study evaluating optimised combination of biological therapy in newly diagnosed high‐risk multiple myeloma and plasma cell leukaemia. BMJ Open. 2021;11(3):e046225. 10.1136/bmjopen-2020-046225 PMC7993167 33762245

[29] Shah JJ , Abonour R , Gasparetto C , et al. Analysis of common eligibility criteria of randomized controlled trials in newly diagnosed multiple myeloma patients and extrapolating outcomes. Clin Lymphoma Myeloma Leuk. 2017;17(9):575‐583.e2. 28886839 10.1016/j.clml.2017.06.013

[30] Richardson PG , San Miguel JF , Moreau P , et al. Interpreting clinical trial data in multiple myeloma: translating findings to the real‐world setting. Blood Cancer J. 2018;8(11):109. 30413684 10.1038/s41408-018-0141-0 PMC6226527

[31] Szabo AG , Iversen KF , Möller S , Plesner T . The clinical course of multiple myeloma in the era of novel agents: a retrospective, single‐center, real‐world study. Clin Hematol Int. 2019;1:220‐228. 34595433 10.2991/chi.d.190805.002 PMC8432372

[32] Verelst SGR , Blommestein HM , De Groot S , et al. Long‐term outcomes in patients with multiple myeloma: a retrospective analysis of the Dutch Population‐based HAematological Registry for Observational Studies (PHAROS). HemaSphere. 2018;2(4):e45. 31723779 10.1097/HS9.0000000000000045 PMC6746001

[33] Durie BGM , Hoering A , Sexton R , et al. Longer term follow‐up of the randomized phase III trial SWOG S0777: bortezomib, lenalidomide and dexamethasone vs. lenalidomide and dexamethasone in patients (Pts) with previously untreated multiple myeloma without an intent for immediate autologous stem. Blood Cancer J. 2020;10(5):53. 32393732 10.1038/s41408-020-0311-8 PMC7214419

[34] Kumar SK , Facon T , Usmani SZ , et al. Updated analysis of daratumumab plus lenalidomide and dexamethasone (D‐Rd) versus lenalidomide and dexamethasone (Rd) in patients with transplant‐ineligible Newly Diagnosed Multiple Myeloma (NDMM): the phase 3 Maia study. Blood. 2020;136(Supplement 1):24‐26.32430494

[35] Cavo M , Gay F , Beksac M , et al. Autologous haematopoietic stem‐cell transplantation versus bortezomib–melphalan–prednisone, with or without bortezomib–lenalidomide–dexamethasone consolidation therapy, and lenalidomide maintenance for newly diagnosed multiple myeloma (EMN02/HO95): a multicentre, randomised, open‐label, phase 3 study. Lancet Haematol. 2020;7(6):e456‐e468. 32359506 10.1016/S2352-3026(20)30099-5

[36] Inoue J , Otsuki T , Hirasawa A , et al. Overexpression of PDZK1 within the 1q12‐q22 amplicon is likely to be associated with drug‐resistance phenotype in multiple myeloma. Am J Pathol. 2004;165(1):71‐81. 15215163 10.1016/S0002-9440(10)63276-2 PMC1618545

