# Supplementary material for: Prognostic gene expression analysis in a retrospective, multinational cohort of 155 multiple myeloma patients treated outside clinical trials
Source: Int J Lab Hematol. 2021 Aug 26;44(1):127–34. doi: 10.1111/ijlh.13691 (PMC9290833; doi:10.1111/ijlh.13691)
Supplement: Supplementary file 1 — Supplementary Material [file IJLH-44-127-s001.docx]

# Supporting Information

**Prognostic and predictive gene expression analysis in a retrospective, multinational cohort of 155 multiple myeloma patients treated outside trials**

Yan-Ting Chen^1^, Erik T. Valent^1^, Erik H. van Beers^1^, Rowan Kuiper^1^, Stefania Oliva^2^, Torsten Haferlach^3^, Wee-Joo Chng^4,5,6^, Martin H. van Vliet^1^, Pieter Sonneveld^7^, and Alessandra Larocca^2^

^1^SkylineDx, Rotterdam, the Netherlands

^2^Myeloma Unit, Division of Hematology, University of Turin, Turin, Italy

^3^MLL Munich Leukemia Laboratory, Munich, Germany

^4^National University Cancer Institute, National University Health System, Singapore, Singapore

^5^Dept of Medicine, Yong Loo Lin School of Medicine, National University of Singapore

^6^Cancer Science Institute of Singapore, National University of Singapore

^7^Department of Hematology, Erasmus MC Cancer Institute, Rotterdam, the Netherlands

**Note:** Supplementary data for this article are available at Clinical Cancer Research Online (http://clincancerres.aacrjournals.org/).

Y.-T. Chen and E. T. Valent contributed equally as first co-authors.

**Corresponding Author:** Martin H. van Vliet, SkylineDx, Brainpark II – Building XI, Lichtenauerlaan 40, 3062ME Rotterdam, The Netherlands. Phone: +31 (0)10 7200310; E-mail: m.vanvliet@skylinedx.com

**Figures**

**
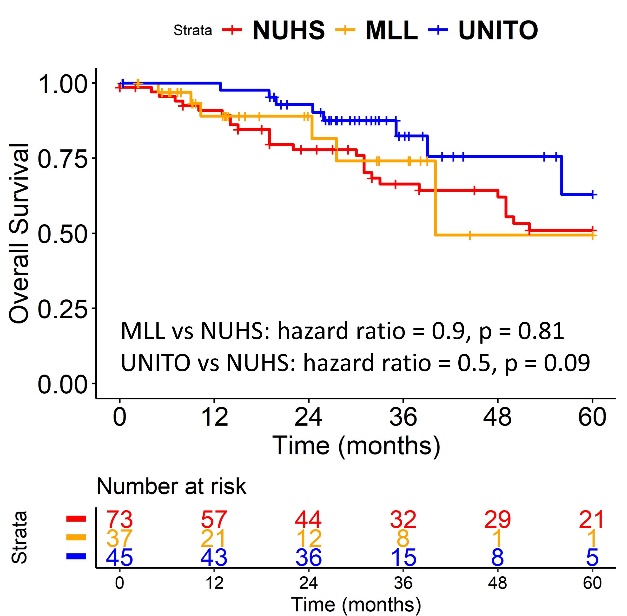
**

**Figure S1:** Kaplan-Meier curves for OS stratified by the three sites where the 155 MM patients were included. The OS was not associated with any specific site.

**
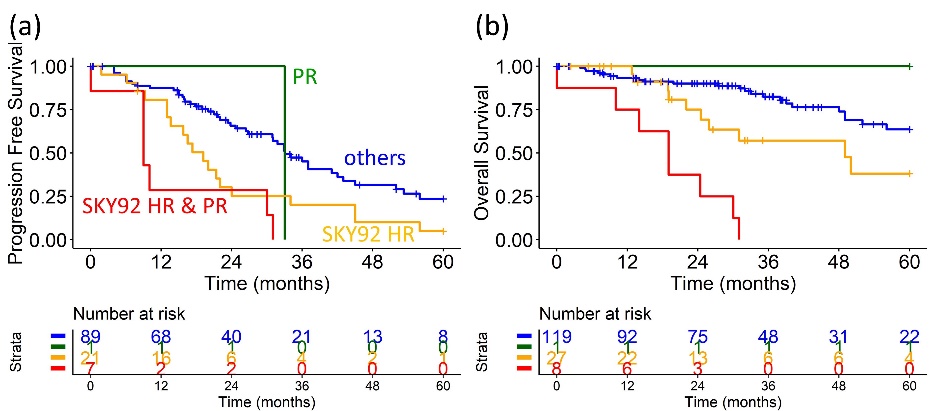
**

**Figure S2:** Kaplan-Meier curves for PFS (A) and OS (B) of patients stratified by both SKY92 and PR-cluster. Since there was only one patient classified as PR positive and SKY92 SR (green), it is unclear whether PR-cluster alone was associated with adverse outcomes. On the other hand, patients in SKY92 HR group (red and orange) were associated with adverse outcomes regardless of the PR-cluster status.

**
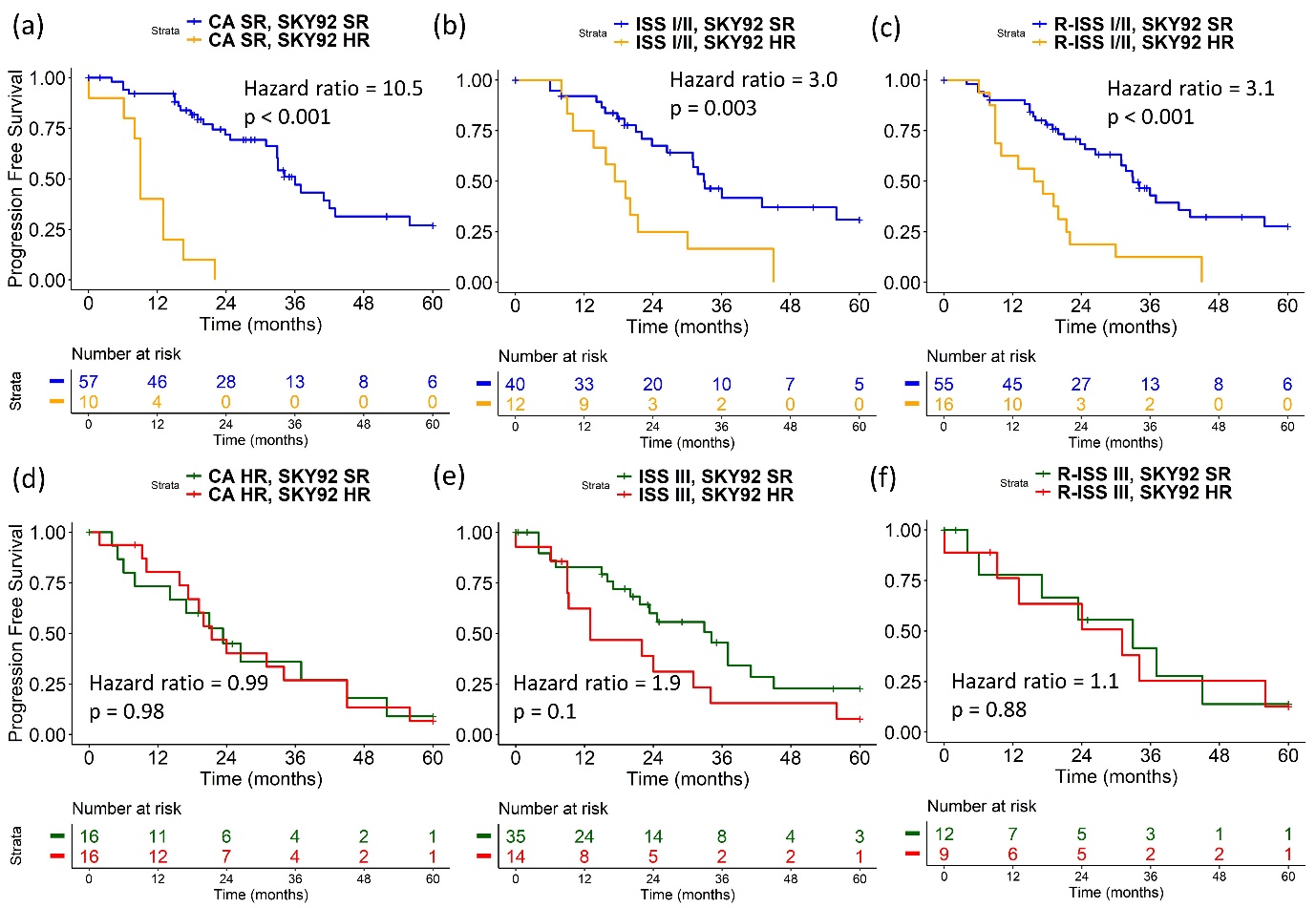
Figure S3:** Kaplan-Meier curves for PFS in the cytogenetic aberrations (CA) standard-risk and high-risk group (A, D), ISS I/II and III (B, E), and R-ISS I/II and III (C, F), with each group being stratified by SKY92. SKY92 found patients with adverse PFS in CA standard-risk (A), ISS I/II (B), R-ISS I/II (C).


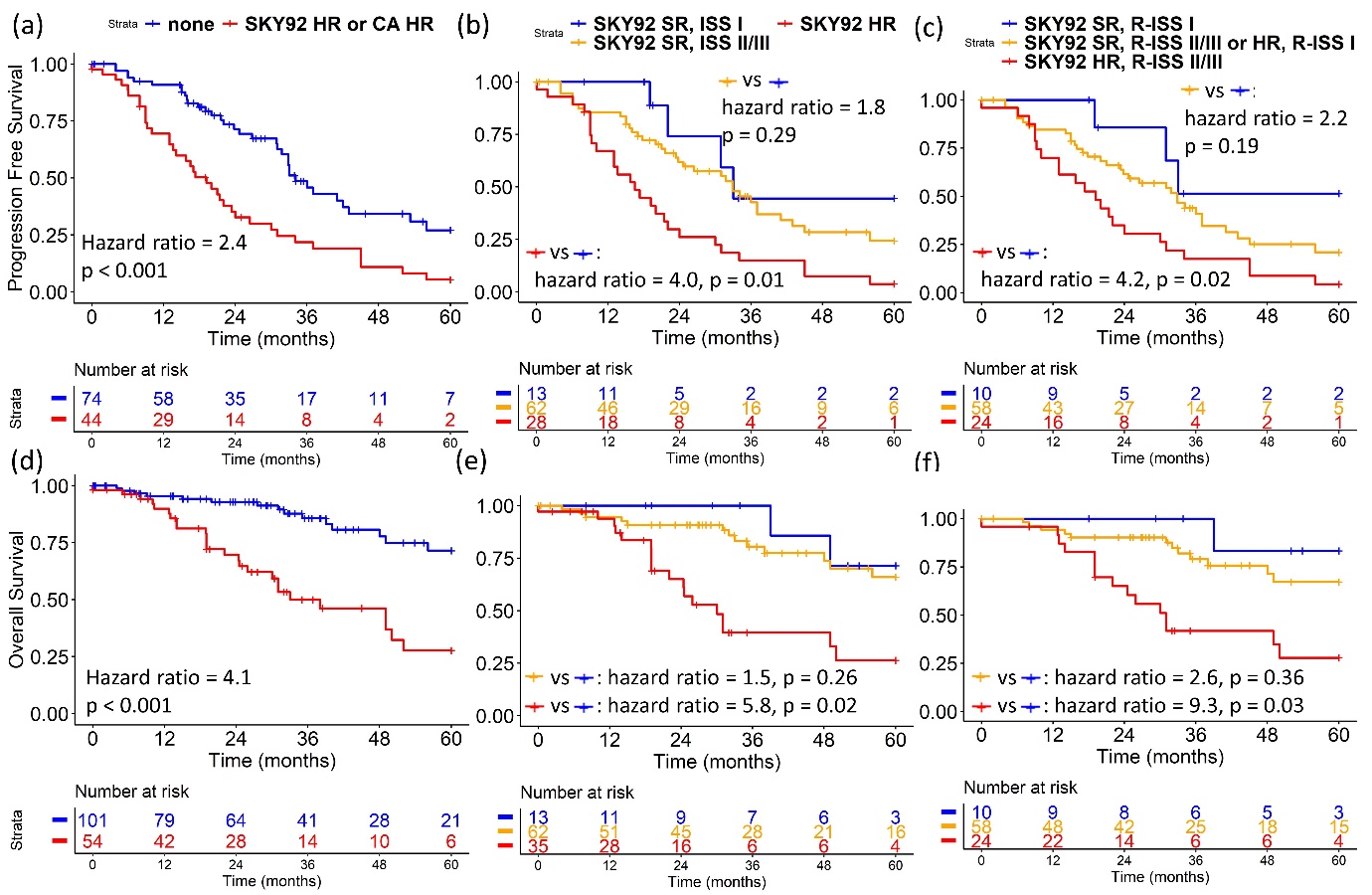


**Figure S4:** Kaplan-Meier curves for OS (A-C) and PFS (D-F) stratified by high-risk cytogenetic aberrations and SKY92 combined (A, D), the combination of ISS with SKY92 (B, E) and R-ISS with SKY92 (C, F). All stratifications had larger hazard ratio with more significance by adding SKY92 compared to the classification of the frequently used markers individually, as depicted previously in Figure 1 (B-D, F-H).

**
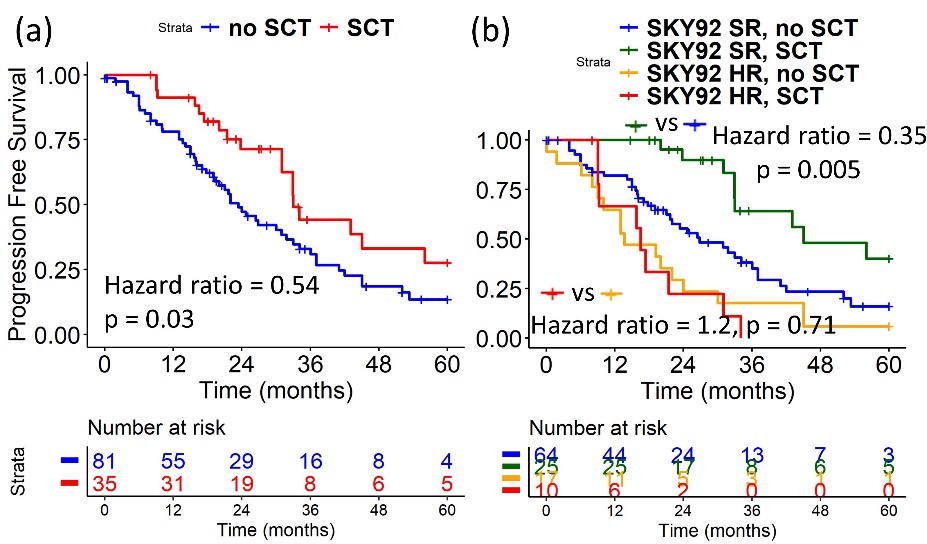
**

**Figure S5:** PFS and OS curves stratified by stem cell transplant (SCT) vs no SCT patients (A and B) and additionally subdivided by the SKY92 biomarker (C and D). The benefit of SCT was only significant in PFS for SKY92 standard risk patients.


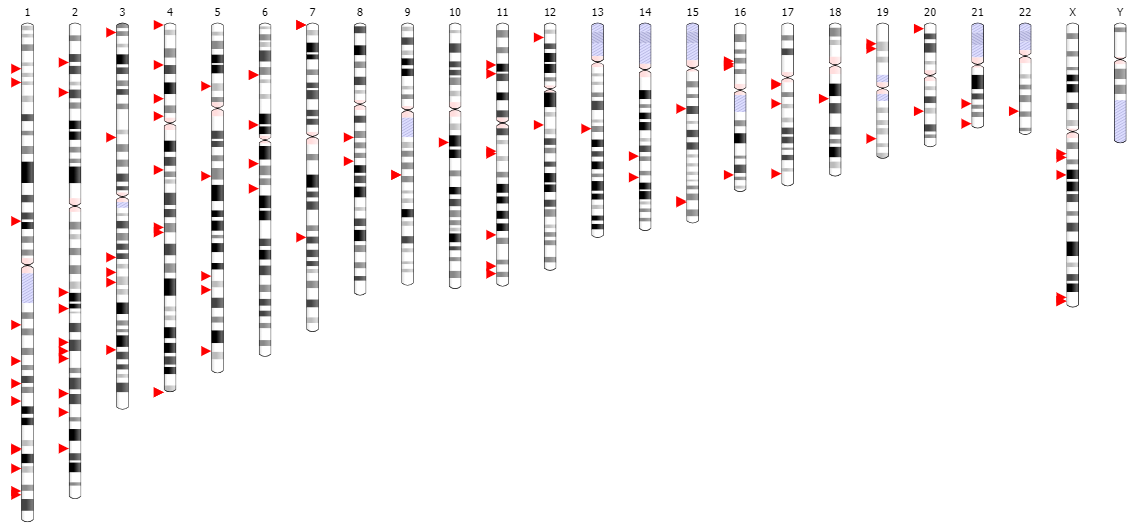


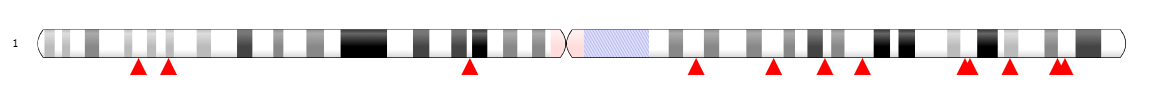


**Figure S6:** SKY92 ideogram showing the 92 genes (red triangles) and specifically highlighting the enrichment for the long arm of chromosome 1. Grey and black bands in each chromosome represent heterochromatin. The pink region is the area around the centromere, and the blue represents variable regions. Generated using Ideogram: <https://eweitz.github.io/ideogram/>

**Tables**

**Table S1:** Validation of the virtual cytogenetic aberrations on the MMprofiler^TM^ assay in comparison to the corresponding interphase values for the health record. Both PPA and NPA were of reasonable accuracies compared to previous results.^24^ TP = true positive, FN = false negative, FP = false positive, TN = true negative, PPA = (TP/(TP+FN)), NPA = (TN/(TN+FP)).

|  | **TP** | **FN** | **FP** | **TN** | **PPA %** | **NPA %** | **unknown** |
| --- | --- | --- | --- | --- | --- | --- | --- |
| t(4;14) | 17 | 0 | 4 | 96 | 100 | 96 | 38 |
| t(11;14) | 22 | 4 | 3 | 89 | 85 | 97 | 37 |
| t(14;16)/t(14;20) | 5 | 1 | 3 | 89 | 83 | 97 | 57 |

**Table S2:** Hazard ratios and p-values for OS and PFS calculated from multivariate Cox proportional hazard models including SKY92, PR-cluster, and cytogenetic high-risk. Significant codes: ** p<0.01, * p<0.05.

|  | **OS** | | **PFS** | |
| --- | --- | --- | --- | --- |
| **Biomarker** | **hazard ratio (95%CI)** | **p-value** | **hazard ratio 95% CI)** | **p-value** |
| SKY92 | 2.9 (1.2 – 6.9) | 0.02^*^ | 2.3 (1.1 – 4.6) | 0.02^*^ |
| PR-cluster | 3.7 (1.5 – 9.1) | 0.004^**^ | 2.5 (1.1 – 5.8) | 0.04^*^ |
| CA high-risk | 1.1 (0.48 – 2.5) | 0.84 | 0.87 (0.45 – 1.7) | 0.69 |

**Table S3:** Survival analysis of OS and PFS analyzed by Cox proportional hazard model. One of the unique features of our non-trial cohort was the diversity of therapies administered to the patients. Because the heterogeneity in treatment, different drugs were aggregated into the drug classes: proteasome inhibitors (PIs), immunomodulatory drugs (IMiDs) and a combination of both PI and IMiD. For this annotation, the scoring over the different drug classes is non-exclusive - since a patient can be treated with multiple drugs in a single regimen. Patients that received a stem cell transplant (SCT) therapy (28%) – which is the standard of care for eligible MM patients - had a longer PFS in the study population, with a HR of 0.54 (p=0.03) for PFS (Figure S5). Of note is this result might reflect the general fact that SCT-eligible patients are younger and fitter than the ineligible population. Although age is an important criterion for SCT eligibility, it was not associated with survival itself. NA = Not available. Significant codes: ** p<0.01, * p<0.05.

|  |  | **OS** | | **PFS** | |
| --- | --- | --- | --- | --- | --- |
| **Therapy** | **n (%)** | **HR (95% CI)** | **p** | **HR (95% CI)** | **p** |
| SCT | 44 (28%) | 0.6 (0.28 – 1.3) | 0.17 | 0.54 (0.32 – 0.93) | 0.03^*^ |
| IMiDs only | 38 (25%) | 1 | - | 1 | - |
| PIs only | 74 (48%) | 0.83 (0.41 – 1.6) | 0.58 | 1.4 (0.82 – 2.4) | 0.22 |
| IMiDs+ PIs | 23 (15%) | 0.41 (0.13 – 1.2) | 0.12 | 0.79 (0.38 – 1.6) | 0.52 |
| Missing | 10 (6%) | NA | NA | NA | NA |

**Table S4:** Case numbers of variables in each ISS stage (n), the percentages of case numbers compared to case numbers in all three stages (%), and confidence (Wilson score) intervals (CI%). The medium age and (minimum-maximum) is shown.

| ISS  Variable | I | | | II | | | III | | | NA |
| --- | --- | --- | --- | --- | --- | --- | --- | --- | --- | --- |
|  | n | % | CI % | n | % | CI % | n | % | CI % | n |
| Transplant | 9 | 30 | (17-48) | 9 | 30 | (17-48) | 12 | 40 | (25-58) | 14 |
| PI | 11 | 23 | (14-37) | 15 | 32 | (20-46) | 21 | 45 | (31-59) | 27 |
| IMiDs | 3 | 9 | (3-24) | 9 | 28 | (16-45) | 20 | 62 | (45-77) | 6 |
| PI+IMiDs | 5 | 28 | (12-51) | 7 | 39 | (20-61) | 6 | 33 | (16-56) | 5 |
| NDMM | 18 | 20 | (13-30) | 27 | 31 | (22-41) | 43 | 49 | (39-59) | 50 |
| RRMM | 2 | 15 | (4-42) | 5 | 38 | (18-64) | 6 | 46 | (23-71) | 4 |
| CA | 6 | 21 | (10-38) | 8 | 28 | (15-46) | 15 | 52 | (34-69) | 10 |
| t(4;14) | 2 | 12 | (3-36) | 4 | 25 | (10-49) | 10 | 62 | (39-82) | 4 |
| t(11:14) | 6 | 38 | (18-61) | 4 | 25 | (10-49) | 6 | 38 | (18-61) | 13 |
| del(17p) | 2 | 29 | (8-64) | 4 | 57 | (25-84) | 1 | 14 | (1-51) | 5 |
| del(13q) | 4 | 13 | (5-29) | 10 | 32 | (19-50) | 17 | 55 | (38-71) | 18 |
| gain(1q) | 2 | 40 | (12-77) | 2 | 40 | (12-77) | 1 | 20 | (1-62) | 11 |
| age | 66  (45-78) |  |  | 63  (46-81) |  |  | 66  (32-90) |  |  | 65  (36-84) |

**Probesets**

**Microarray probes used to define markers SKY92, virtual t(4;14), t(11;14) and t(14;16)/t(14;16), and MM clusters are listed below.**

**Probes for SKY92**

204379_s_at, 202728_s_at, 239054_at, 202842_s_at, 213002_at, 210334_x_at, 201795_at, 38158_at, 208232_x_at, 201307_at, 226742_at, 205046_at, 204026_s_at, 226218_at, 217824_at, 233399_x_at, 224009_x_at, 215177_s_at, 202532_s_at, 238662_at, 212788_x_at, 220351_at, 202542_s_at, 243018_at, 209683_at, 212282_at, 208967_s_at, 225366_at, 217852_s_at, 225601_at, 231210_at, 214482_at, 208942_s_at, 219550_at, 231989_s_at, 202553_s_at, 223811_s_at, 221041_s_at, 221677_s_at, 213350_at, 200775_s_at, 226217_at, 217728_at, 201930_at, 216473_x_at, 211714_x_at, 221755_at,

AFFX-HUMISGF3A/M97935_MA_at, 206204_at, 217548_at, 215181_at, 217732_s_at, 214612_x_at, 202813_at, 200875_s_at, 201292_at, 222680_s_at, 233437_at, 223381_at, 209026_x_at, 221606_s_at, 231738_at, 230034_x_at, 213007_at, 242180_at, 202322_s_at, 208904_s_at, 214150_x_at, 238116_at, 208732_at, 200701_at, 208667_s_at, 208747_s_at, 218662_s_at, 211963_s_at, 201555_at, 207618_s_at, 200933_x_at, 221826_at, 218355_at, 219510_at, 218365_s_at, 222713_s_at, 222154_s_at, 228416_at, 201102_s_at, 203145_at, 238780_s_at, 202884_s_at, 201398_s_at, 212055_at, 202107_s_at

**Probes for t(4;14)**

222777_s_at, 222778_s_at, 212148_at, 217867_x_at, 223822_at, 222258_s_at, 227290_at, 221261_x_at, 227434_at, 212813_at, 212151_at, 227084_at, 211709_s_at, 227692_at, 223313_s_at, 223472_at, 204379_s_at, 205830_at, 205131_x_at

**Probes for t(11;14)**

208711_s_at, 208712_at, 235518_at

**Probes for t(14;16)/t(14;20)**

204589_at, 205286_at

**Probes for MM clusters**

204409_s_at, 204379_s_at, 200953_s_at, 228592_at, 201005_at, 208712_at, 230493_at, 208711_s_at, 210432_s_at, 200602_at, 217901_at, 206574_s_at, 213566_at, 224724_at, 228599_at, 232231_at, 206760_s_at, 222943_at, 235228_at, 206045_s_at, 204439_at, 223823_at, 242100_at, 205590_at, 212843_at, 230781_at, 218589_at, 206385_s_at, 204602_at, 207739_s_at, 1554242_a_at, 208235_x_at, 219895_at, 206640_x_at, 213155_at, 205898_at, 209695_at, 204489_s_at, 222453_at, 205830_at, 210587_at, 227697_at, 212190_at, 221097_s_at, 242625_at, 232352_at, 205114_s_at, 203153_at, 235278_at, 213992_at, 212063_at, 204014_at, 210993_s_at, 236646_at, 230425_at, 214156_at, 229580_at, 206991_s_at, 217867_x_at, 211709_s_at, 211517_s_at, 220068_at, 220850_at, 218223_s_at, 233555_s_at, 244780_at, 235518_at, 217418_x_at, 219954_s_at, 205286_at, 204469_at, 202391_at, 231963_at, 207086_x_at, 215051_x_at, 202946_s_at, 213110_s_at, 217889_s_at, 205124_at, 206759_at, 207663_x_at, 217963_s_at, 208158_s_at, 202207_at, 210356_x_at, 210916_s_at, 228523_at, 233059_at, 227949_at, 205559_s_at, 236065_at, 204416_x_at, 1555756_a_at, 213924_at, 229221_at, 229450_at, 226702_at, 212771_at, 201743_at, 235377_at, 222439_s_at, 223374_s_at, 201721_s_at, 1563209_a_at, 224851_at, 203186_s_at, 202011_at, 208155_x_at, 239468_at, 1557905_s_at, 204760_s_at, 204066_s_at, 205442_at, 235735_at, 209901_x_at, 228737_at, 242344_at, 227372_s_at, 235400_at, 233500_x_at, 202145_at, 222108_at, 220595_at, 212148_at, 208373_s_at, 243780_at, 220645_at, 1558185_at, 201720_s_at, 205789_at, 217744_s_at, 229552_at, 209555_s_at, 208358_s_at, 216430_x_at, 227180_at, 227394_at, 213095_x_at, 205309_at, 222062_at, 1554485_s_at, 219837_s_at, 206150_at, 238546_at, 220005_at, 200923_at, 204959_at, 212446_s_at, 211032_at, 1557257_at, 220059_at, 209183_s_at, 213032_at, 227692_at, 210715_s_at, 205122_at, 223940_x_at, 228956_at, 204999_s_at, 228532_at, 206978_at, 202552_s_at, 204490_s_at, 1558733_at, 235666_at, 209847_at, 204589_at, 213693_s_at, 204328_at, 202075_s_at, 228284_at, 212686_at, 206023_at, 207194_s_at, 231887_s_at, 231899_at, 215565_at, 214230_at, 219370_at, 227336_at, 241844_x_at, 226325_at, 226342_at, 206488_s_at, 213050_at, 200648_s_at, 228729_at, 203559_s_at, 214329_x_at, 227266_s_at, 212151_at, 210762_s_at, 204971_at, 202554_s_at, 204749_at, 200762_at, 1566433_at, 221698_s_at, 222281_s_at, 227341_at, 219243_at, 213797_at, 222810_s_at, 214464_at, 219403_s_at, 206111_at, 206950_at, 227510_x_at, 244632_at, 229309_at, 238282_at, 222392_x_at, 221648_s_at, 213446_s_at, 235657_at, 216840_s_at, 220145_at, 205578_at, 223822_at, 222365_at, 237737_at, 220253_s_at, 235802_at, 230134_s_at, 230550_at, 205862_at, 242832_at, 201137_s_at, 200989_at, 219440_at, 206420_at, 226560_at, 209118_s_at, 208079_s_at, 205936_s_at, 204802_at, 222258_s_at, 205123_s_at, 228827_at, 211794_at, 219667_s_at, 211986_at, 1559975_at, 211434_s_at, 208961_s_at, 203423_at, 1552553_a_at, 209627_s_at, 203641_s_at, 1558678_s_at, 202743_at, 1553226_at, 229584_at, 235645_at, 213519_s_at, 1557030_at, 203304_at, 227353_at, 203875_at, 241703_at, 217878_s_at, 209684_at, 1569136_at, 227742_at, 228766_at, 242134_at, 204126_s_at, 205529_s_at, 213324_at, 228450_at, 203751_x_at, 226864_at, 209708_at, 209966_x_at, 201101_s_at, 243310_at, 202687_s_at, 1557780_at, 223204_at, 209949_at, 206314_at, 232278_s_at, 1558186_s_at, 206059_at, 202786_at, 235372_at, 205027_s_at, 216452_at, 203213_at, 1553105_s_at, 205230_at, 209619_at, 227410_at, 203479_s_at, 214708_at, 230192_at, 211795_s_at, 222876_s_at, 205560_at, 235593_at, 1553043_a_at, 202973_x_at, 213506_at, 213817_at, 205352_at, 222777_s_at, 228094_at, 219093_at, 1564310_a_at, 209062_x_at, 227799_at, 227067_x_at, 227817_at, 221880_s_at, 205718_at, 1556583_a_at, 244137_at, 227036_at, 227915_at, 217999_s_at, 229308_at, 202688_at, 241752_at, 1554474_a_at, 222670_s_at, 221122_at, 202643_s_at, 204641_at, 220770_s_at, 210145_at, 205909_at, 201951_at, 1564154_at, 226545_at, 200986_at, 232125_at, 209201_x_at, 239135_at, 205686_s_at, 238332_at, 213484_at, 200671_s_at, 231595_at, 1554406_a_at, 239205_s_at, 226980_at, 228055_at, 206503_x_at, 36711_at, 204122_at, 238633_at, 205098_at, 207638_at, 221843_s_at, 241396_at, 205640_at, 1557383_a_at, 229084_at, 204731_at, 230748_at, 217818_s_at, 203148_s_at, 204040_at, 236198_at, 211085_s_at, 226479_at, 217542_at, 230399_at, 1559072_a_at, 230012_at, 218928_s_at, 218723_s_at, 239823_at, 210232_at, 241762_at, 223932_at, 204472_at, 209828_s_at, 217234_s_at, 209381_x_at, 234980_at, 222962_s_at, 218815_s_at, 224771_at, 221582_at, 219983_at, 209626_s_at
